# 

**Published:** 1899-09-09

**Authors:** 


					THE HOSPITAL. tl The standard of highest purity."?Lancet. September 9th, 1899.
CADBURY'S rf* H CADBURY'S
COOOA m> M m Jig COCOA
msolot.lt ran. <-> no druo? or alIalie. uho
JLa'scoiy Western Infirmary
No. 676.Vfo>1. XXVJ. Saturday,
Sept. 9th, 1899.
"Subscription Terms,"]
see p. 400. J
wRWnrLK~AM XJ/?MVCH HbBMlTAL
PRICE TWOPENCE.

				

